# Placental Hofbauer cells assemble and sequester HIV-1 in tetraspanin-positive compartments that are accessible to broadly neutralizing antibodies

**DOI:** 10.7448/IAS.18.1.19385

**Published:** 2015-01-22

**Authors:** Erica L Johnson, Hin Chu, Siddappa Nagadenahalli Byrareddy, Paul Spearman, Rana Chakraborty

**Affiliations:** 1 Department of Pediatrics, Emory University School of Medicine Atlanta, GA, USA; 2 Department of Pathology & Laboratory Medicine, Emory University School of Medicine Atlanta, GA, USA; 3 Emory Vaccine Center, Emory University School of Medicine Atlanta, GA, USA

**Keywords:** placenta, Hofbauer cells, HIV-1, VCCs, bNAbs, MTCT

## Abstract

**Introduction:**

Within monocyte-derived macrophages, HIV-1 accumulates in intracellular virus-containing compartments (VCCs) that are inaccessible to the external environment, which implicate these cells as latently infected HIV-1 reservoirs. During mother-to-child transmission of HIV-1, human placental macrophages (Hofbauer cells (HCs)) are viral targets, and have been shown to be infected *in vivo* and sustain low levels of viral replication *in vitro*; however, the risk of *in utero* transmission is less than 7%. The role of these primary macrophages as viral reservoirs is largely undefined. The objective of this study is to define potential sites of viral assembly, accumulation and neutralization in HCs given the pivotal role of the placenta in preventing HIV-1 infection in the mother-infant dyad.

**Methods:**

Term placentae from 20 HIV-1 seronegative women were obtained following caesarian section. VCCs were evaluated by 3D confocal and electron microscopy. Colocalization R values (Pearson's correlation) were quantified with colocalization module of Volocity 5.2.1. Replication kinetics and neutralization studies were evaluated using p24 ELISA.

**Results:**

We demonstrate that primary HCs assemble and sequester HIV-1_BaL_ in intracellular VCCs, which are enriched in endosomal/lysosomal markers, including CD9, CD81, CD63 and LAMP-1. Following infection, we observed HIV-1 accumulation in potentially acidic compartments, which stained intensely with Lysotracker-Red. Remarkably, these compartments are readily accessible via the cell surface and can be targeted by exogenously applied small molecules and HIV-1-specific broadly neutralizing antibodies. In addition, broadly neutralizing antibodies (4E10 and VRC01) limited viral replication by HIV-1-infected HCs, which may be mediated by FcγRI.

**Conclusions:**

These findings suggest that placental HCs possess intrinsic adaptations facilitating unique sequestration of HIV-1, and may serve as a protective viral reservoir to permit viral neutralization and/or antiretroviral drug entry *in utero*.

## Introduction

The placenta is characterized by close contact between maternal decidua and invading foetal-derived chorionic villi. The chorionic villus is lined by trophoblast and contains a connective core of foetal blood vessels and numerous placental macrophages (Hofbauer cells (HCs)). A number of studies have documented ongoing trafficking of maternal immune cells *in utero*, through the placenta into foetal blood [1–3]. In addition, it is well established that humoral immunity can be passively transferred from mother to baby, prenatally across the placenta. During maternal HIV-1 infection, this transfer across the placenta may include maternal neutralizing antibodies (NAbs) and virions (free, cell or Ab-associated), which interact directly with HCs prior to entering the foetal circulation [4]. Interestingly, HCs express the HIV-1 (co)-receptors CD4, CCR5, CXCR4 and DC-SIGN on their cell surface along with Fcγ receptors which can sequester Abs and Ab-virion immune complexes [5]. In spite of this potentially permissive phenotype, the risk of *in utero* transmission is only 7%, which may implicate HCs as important mediators of protection during ongoing HIV-1 exposure.

We previously demonstrated that HCs limit HIV-1 replication *in vitro* by induction of immunoregulatory cytokines [6]. However, the sites of viral assembly and accumulation are uncharacterized in HCs, along with the nature of potential virus-containing compartments (VCCs). HIV-1 assembly and release occurs in T cells at the plasma membrane [7–9], while HIV-1-infected peripheral blood macrophages accumulate large vacuoles holding infectious virions [10,11]. This endosomal compartment forms intraluminal vesicles marked by multi-vesicular bodies, characteristic markers of which include CD81, CD9, MHC Class II and CD63 [12,13]. It has been reported that macrophages harbour infectious HIV-1 over a prolonged period [14] and that the virus has evolved strategies to prevent viral degradation [10]. We have previously shown that VCCs in peripheral blood macrophages are effectively closed compartments, inaccessible to the external environment [13], which may protect from recognition by antibodies and prevent neutralization or attachment of binding non-NAbs. Although a matter of debate, these data underscore a potential cell-specific role for a specialized compartment in HIV-1 assembly and accumulation.

Here we characterize VCCs in HIV-1_BaL_-infected placental HCs and demonstrate viral accumulation within intracellular vesicles. These compartments are specifically labelled by CD9 and CD81, and the majority of these endosomal compartments appear to be acidic. These tetraspanin-rich compartments can be accessed by exogenously applied small molecules, along with HIV-1-specific broadly neutralizing antibodies (bNAbs), VRC01 (gp120-directed) and 4E10 (gp41-directed), which are largely dependent on interaction with FcγRI (CD64). Defining potential sites of viral assembly, accumulation and neutralization in HIV-1 (co)-receptor-positive HCs is important in identifying transmission dynamics and correlates of protection to HIV-1 given the pivotal role of the placenta in offsetting *in utero* HIV-1 infection.

## Methods

### Ethics statement

With written informed consent, term placentae (>37 weeks gestation) from 20 HIV-1/hepatitis B seronegative women were obtained following caesarian section from Emory Midtown Hospital in Atlanta, GA. Study approval was granted from Emory University Institutional Review Board (IRB). Peripheral blood was obtained from healthy adult volunteers according to a protocol approved by the Emory University IRB. Written informed consent was obtained from all donors.

### Isolation and culture of HCs and monocyte-derived macrophages

To isolate HCs, the decidua basalis was dissected from the placenta, as previously described [6]. Briefly, the tissue was washed, minced and resuspended in medium containing 10% trypsin/EDTA (Sigma Chemical Co., St. Louis, MO), followed by resuspension in media containing 1 mg/ml collagenase IV (Sigma), 10 U/ml dispase (Worthington Biochemical Corp., Lakewood, NJ) and 0.2 mg/ml of DNAse I (Sigma). The digested tissue passed through a 70 µm cell strainer (BD Biosciences, San Jose, CA). The mononuclear cells were isolated by density gradient centrifugation, and CD14^+^ Magnetic Cell Sorting was performed using anti-CD14 magnetic beads (Miltenyi Biotech, Auburn, CA). For monocyte-derived macrophages (MDMs), monocytes were isolated from buffy coats of peripheral blood donors by density gradient centrifugation prior to positive selection for CD14 (Miltinyi). The cells were cultured with GM-CSF for seven days for MDM differentiation.

### Antibodies and immunostaining reagents

Mouse monoclonal antibodies against CD9, CD81, CD63, CD64 and LAMP-1 were obtained from BD Biosciences (San Jose, CA); and mouse monoclonal antibody against Transferrin Receptor (TfR) was obtained from Abcam Inc. (Cambridge, MA). HIV-1-Gag detection was performed with mouse anti-p24 monoclonal CA-183 [15] or mouse anti-p24-FITC (KC57-FITC, Beckman Coulter, Fullerton, CA). Alexa Fluor-mouse anti-human, -goat anti-mouse, -goat anti-rabbit and -rabbit anti-goat secondary antibodies, as well as the DAPI (4',6-diamidino-2-phenylindole) were obtained from Molecular Probes (Eugene, OR). IgG1 κ human monoclonals 4E10 (directed against gp41) and VRC01 (gp120) were provided by the AIDS Reference Reagent Program.

### Virus stocks and infections

HCs were incubated with HIV-1_BaL_ virus stock at one 50% tissue culture infectious dose (TCID_50_) per cell overnight. Cells were then washed with media and incubated for 0–6 days before harvesting for analysis.

### Immunofluorescence microscopy

HCs and MDMs were fixed in 4% paraformaldehyde. Cells were then permeabilized with 0.2% Triton X-100 and blocked in Dako blocking buffer. Cells were stained with primary then secondary antibodies. DAPI was used to stain the nuclei of the cells. The coverslips were mounted in Gelvatol. To label HCs prior to permeabilization, cells were immunolabelled with primary antibodies against tetraspanins at 4°C and 37°C, and then fixed. Labelled cells were then permeabilized, blocked and stained for secondary antibody for the tetraspanins and Gag as described above.

### Electron microscopy

HIV-1-infected HCs were fixed with 2.5% glutaraldehyde in 0.1 M cacodylate buffer (pH 7.4) at 4°C, and then washed. The cells were then post-fixed with 1% osmium tetroxide and 1.5% potassium ferrocyanide in 0.1 M cacodylate buffer. The cells were dehydrated and then embedded in Eponate 12 resin. Ultrathin sections were cut on an RMC PowerTome XL ultramicrotome at 70 nm, stained with 5% aqueous uranyl acetate and 2% lead citrate, and examined on a JEOL IEM-1400 transmission electron microscope equipped with Gatan UltraScan US1000.894 and Orius SC1000.832 CCD cameras.

### Low-molecular-weight dextran accessibility

HCs were infected with HIV-1_BaL_ for three days and treated with 0.5 mg/ml lysine fixable Texas Red Dextran (Dex-TR, 3000 MW, Molecular Probes, Eugene, OR). For studies at 37°C, HCs were washed, followed by adding pre-warmed media containing 0.5 mg/ml Dex-TR. The cells were incubated at 37°C for 30 minutes. Dex-TR labelled HCs were then washed with PBS and fixed with 4% paraformaldehyde. For studies at 4°C, HCs were first cooled on ice. The cells were washed, followed by adding cold media containing 0.5 mg/ml Dex-TR. The cells were then incubated at 4°C for 30 minutes. Dex-TR labelled HCs were fixed with 4% paraformaldehyde. Fixed HCs were immunolabelled for HIV-1 Gag as described.

### Neutralization assays

All neutralization assays were performed as described previously [16–18] with few modifications. Briefly, HCs were infected overnight, prior to addition of HIV-1-specific antibodies (10 µg/ml). Controls include media alone and human IgG. For antibody competition experiments, HCs were incubated for 30 minutes with 10 µg/ml of purified anti-FcγRI monoclonal IgG prior to NAb experiments. For all conditions, free virus and antibodies were removed on day 4 by washing. Cells were then cultured until day 10, and p24 was measured in supernatant by ELISA (Advance Bioscience Laboratory Inc., Rockville, MD).

### Image and statistical analysis

The imaging Deltavision system (Applied Precision) was equipped with an Olympus IX70 microscope and a CoolSnap HQ2 digital camera. Imaging processing/de-convolution was performed using softWoRx 3.7.0. Colocalization *R* values (using Pearson's correlation) were quantified with Volocity 5.5.1. Volocity, Adobe Photoshop CS5.1 and Adobe Illustrator CS6 were used to analyze and adjust the images. The images were never modified apart from enhancing brightness. Statistical analysis was performed using the SigmaPlot 12 software package.

## Results

### HCs exhibit a distinct and variable morphology in culture

**Figure 1:**
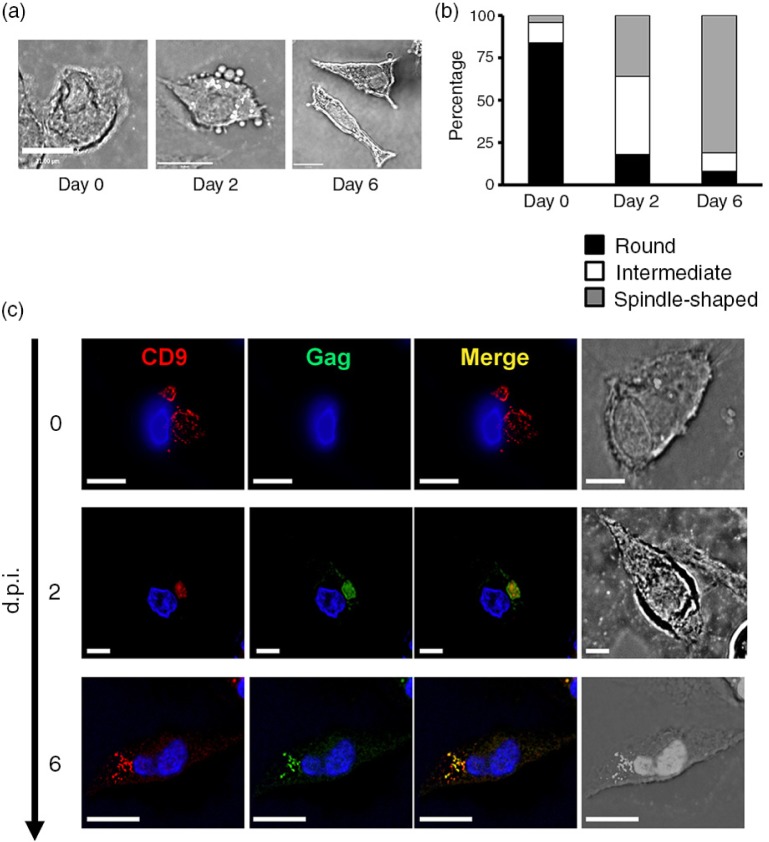
HCs exhibit a distinct and variable morphology in culture. HCs were maintained in complete medium over six days, with or without HIV-1_BaL_ infection. The morphology of uninfected HCs were analyzed over time and presented as (a) bright field images, and (b) the proportion of each phenotype is displayed. Scale bar in panel a*=*11 µm. To analyze the subcelluar localization of HIV-1 in HCs, at day 0, along with day 2 and 6 post-infection, cells were fixed, permeabilized and labelled with primary antibodies against HIV-1 Gag (green, anti-CA183) and CD9 (red, anti-CD9) (c). Scale bar in panel c for day 0 and day 2=4.30 µm. Scale bar in panel c for day 6=11 µm. Image acquisition was performed with an Applied Precision Deltavision deconvolution microscope. Sections and bars shown represent a minimum of 30 cells for each condition from 10 donors.

HCs were maintained in complete medium over six days. HCs were noted to be 10–20 µm in size and displayed a pleiomorphic phenotype. In uninfected and HIV-1-infected HCs, their shape changed from round vacuolated cells on day 0 to a partially elongated spindle-like appearance over six days (Figure 1a and 1b), characteristic of HCs within the placental villi [19,20]. These cells can evolve from macrophage-like to a unique fibroblast-like morphology [21].

Prior to infection, uninfected HCs and MDMs are similar and both have the characteristic CD9 (tetraspanin) intracellular compartment found typically in macrophages (Figure 1c) [13]. A well-documented feature of HIV-1-infected MDMs includes the presence of VCCs, which reveal intense intracellular accumulations of virions in the CD9 compartment (Supplementary file 1). Here we show in HCs on two and six days post-HIV-1 infection, Gag and CD9 displayed strong intracellular colocalization, similar to MDMs, despite the change in morphology (Figure 1c).

### In HIV-1-infected HCs, virus assembles in intracellular tetraspanin-rich compartments

**Figure 2:**
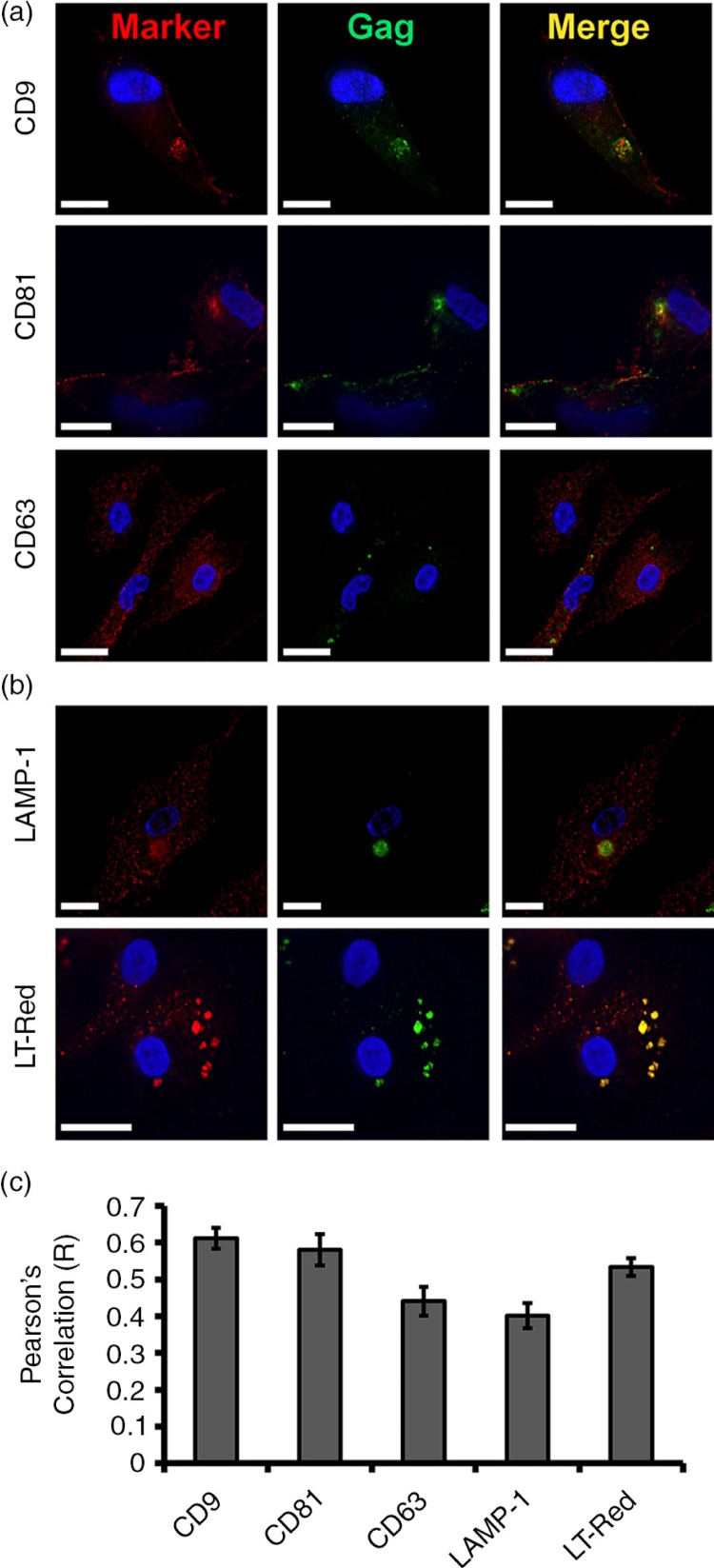
In HIV-1-infected HCs, virus assembles in intracellular tetraspanin-rich compartments. Human Hofbauer cells infected with HIV-1_BaL_ were analyzed by confocal immunofluorescence microscopy three days post-infection. Cells were fixed, permeabilized and labelled with primary antibodies against HIV-1 Gag (green, anti-CA183), (a) tetraspanins (red, anti-CD9, anti-CD81, and anti-CD63) and (b) anti-LAMP-1 (red). (b) Cells were also incubated at 37°C with Lysotracker Red (LT-Red) and fixed, permeabilized and labelled with primary antibodies against HIV-1 Gag (green, anti-CA183). Scale bar=9.0 µm. (c) HIV-1 Gag colocalization and partial colocalization *R* values (using Pearson's correlation) were quantified with the colocalization module of Volocity 5.2.1. Image acquisition was performed with an Applied Precision Deltavision deconvolution microscope. Sections shown represent a minimum of 30 cells for each condition from 10 donors. Data in bar graphs are expressed as the mean+SE (*R* value) of triplicate sections from 10 donors. Asterisks (*p<0.01) indicate values that are significantly higher when compared to TfR (control).

Tetraspanin microdomains have been proposed as sites of HIV-1 assembly [22,23]. To characterize the VCCs in HIV-1-infected HCs, we examined the distribution of CD9, CD81 and CD63. Immunofluorescence revealed staining of intracellular structures in association with Gag. The distribution of CD9 and CD81 differed from CD63 (Figure 2a). VCCs in HIV-1-infected HCs were strongly labelled for CD9 and CD81. In comparison to CD63, immunofluorescence revealed distribution with a tubulo-vesicular pattern similar to tubular lysosomes as characterized for mouse macrophages [24]. Next HCs were immunolabelled with lysosomal markers LAMP-1 and Lysotracker-Red (LT-Red). LT-Red is a cationic fluorescent dye that preferentially accumulates in acidic organelles. LAMP-1 and LT-Red labelling was noted within a network of vesicles throughout the HC in addition to the VCCs (Figure 2b). To control for antibody specificity, we repeated all immunofluorescence experiments with monoclonal anti-TfR. The TfR is an unrelated protein and does not colocalize with Gag in HCs (Supplementary file 2) or MDMs [25]. We noted HIV-1 Gag colocalization *R* values of 0.61+0.02, 0.58+0.04, 0.44+0.03, 0.40+0.03, 0.53+0.05 and 0.2+0.03 for CD9, CD81, CD63, LAMP-1, LT-Red and TfR, respectively (Figure 2c and Supplementary file 2). This suggests that CD9 and CD81 labelling is more specific to VCCs within HCs.

### Electron microscopy of HIV-1-infected HCs reveals viral budding at the plasma membrane and within intracellular compartments

**Figure 3:**
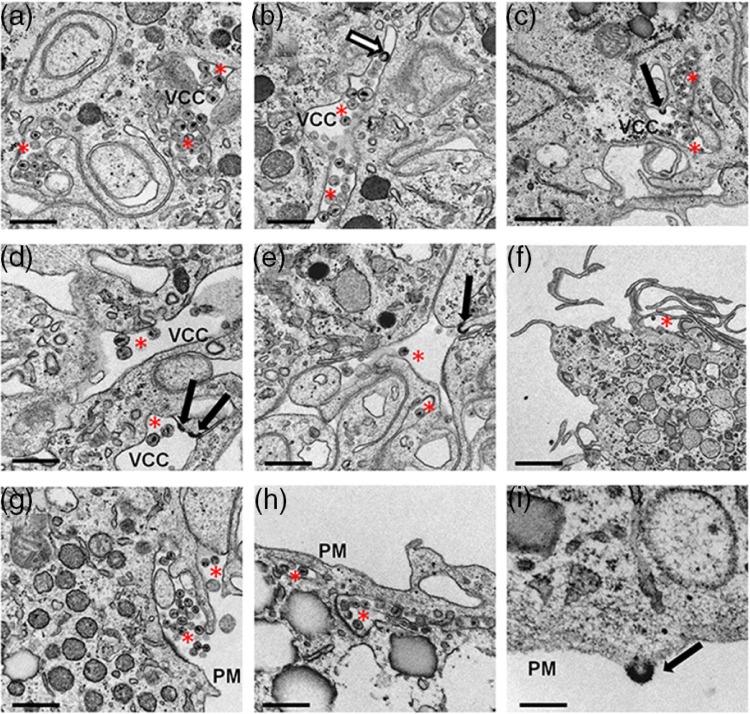
Electron microscopy of HIV-1 infected HCs reveals viral budding at plasma membrane and within intracellular compartments. HIV-1_BaL_ infected HCs were fixed and analyzed by standard electron microscope processing procedures five days post-infection. Sections show intracellular virus-containing compartments (VCCs) with mature virions (red asterisk, a–h), budding profiles (solid arrows, c–e, i) and immature virions (open arrows, b). Mature viral particles were seen under folds (f and h) of the plasma membrane (PM) and in the extracellular space adjacent to the PM (g). Virus budding profiles were observed on the PM (i). Bars represent 0.6 µm for a, b, e and g; 1.5 µm for c and f; 0.6 µm for d and h; and 0.3 µm for i. Sections shown represent a minimum of 20 cells for each condition from four donors.

In T cells and several non-hematopoietic cell lines, the majority of virus particles assemble at the cell surface, but in primary MDMs these events occur almost entirely in intracellular VCCs [10,26]. HCs infected with HIV-1_BaL_ for five days were fixed for transmission electron microscopy (Figure 3). The cells were infected for a longer time period to increase intracellular viral stores. Accumulation of mature HIV-1 virions was readily detected within intracellular VCCs throughout the cytoplasm, near the plasma membrane and in the extracellular space adjacent to the plasma membrane. Early-/late-budding viral particles were frequently detected within the VCCs and on the plasma membrane, suggesting that HIV-1 virions are assembled in these compartments.

### Low-molecular-weight dextrans can access the VCCs in HIV-1-infected HCs

**Figure 4:**
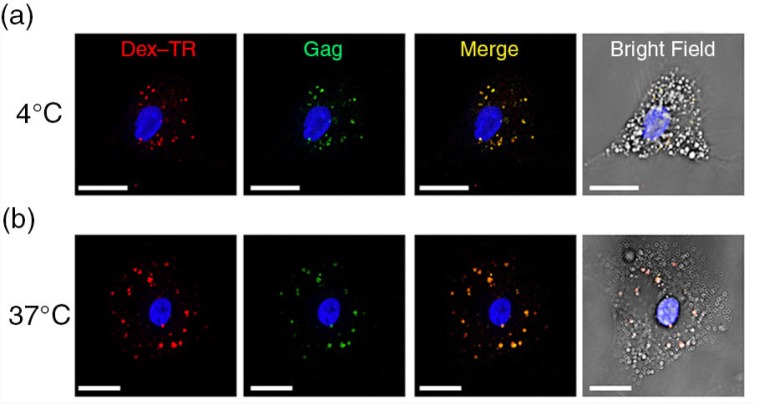
Low-molecular-weight dextrans can access the VCCs in HIV-1-infected HCs. HIV-1-infected HCs were incubated with Texas Red Dextran, 3000 MW, at 4°C or 37°C for 30 minutes. Cells were then fixed and stained for Gag (green). (a) Representative images of cells incubated at 4°C. (b) Representative image of cells incubated at 37°C. Scale bar=11 µm. Sections shown represent a minimum of 30 cells for each condition from 10 donors.

We examined the ability of fluorescent low-molecular-weight dextran to access the VCC in unfixed HCs. HIV-1-infected HCs were incubated at 4°C or 37°C with Texas Red Dextran (Dex-TR, 3000 MW) prior to fixation, permeabilization and staining as before. Staining at 4°C to reduces membrane movement and phagocytosis, while labelling at 37°C allows for active uptake. HCs incubated at 4°C and 37°C demonstrated strong colocalization of dextran and Gag in intracellular compartments (Figure 4a and 4b). These results indicate that the majority of VCCs are accessible to the external environment.

### Internal VCCs within HCs are accessible to antibodies

**Figure 5:**
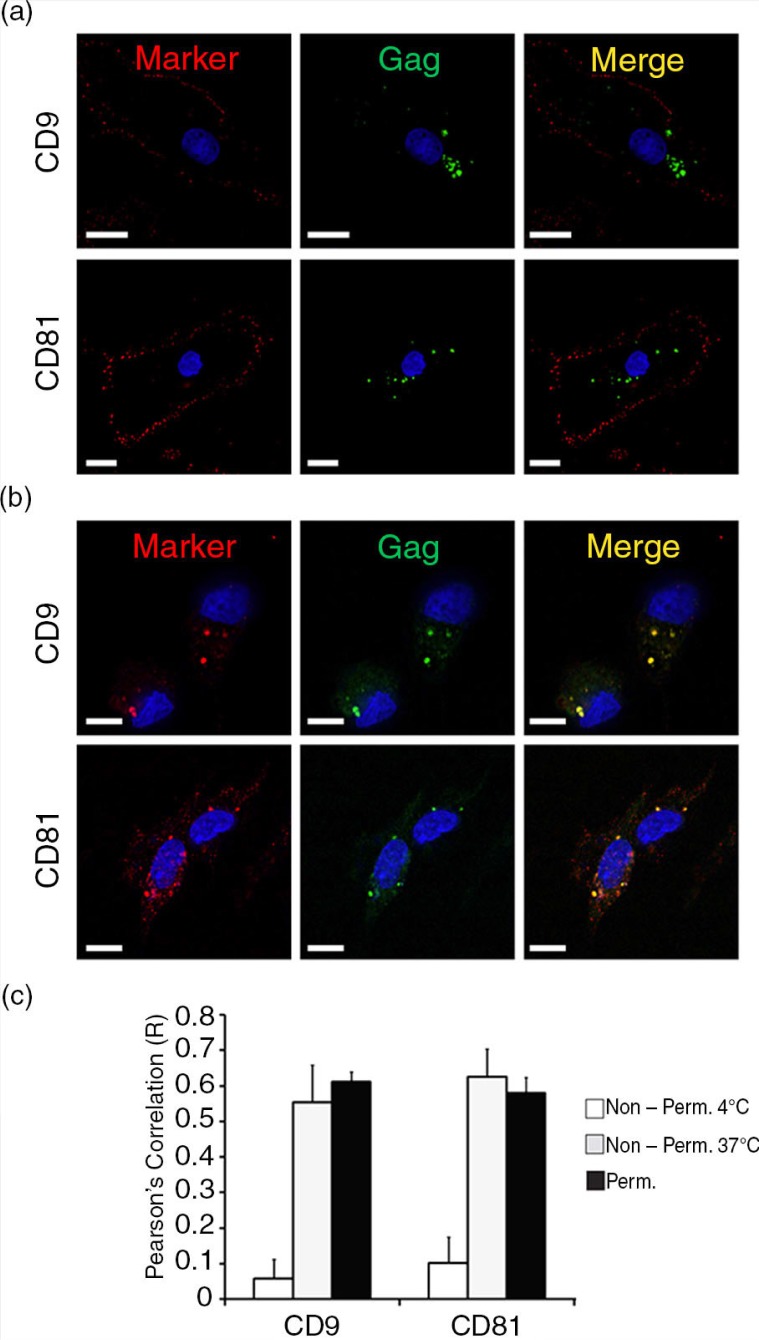
HC internal VCCs are accessible to antibodies. Human HCs were infected with HIV-1_BaL_ for three days. Infected HCs were immunolabelled at 4°C (a) or 37°C (b) prior to fixation/permeabilization and labelled with primary antibodies against tetraspanins (red, anti-CD9 and anti-CD81). Labelled HCs were then permeabilized and immunolabelled for HIV-1 Gag (green, anti-CA183). Image acquisition was performed with an Applied Precision Deltavision deconvolution microscope. Sections shown represent a minimum of 30 cells for each condition from 10 donors. Scale bar=7 µm. (c) HIV-1 Gag colocalization and partial colocalization *R* values (using Pearson's correlation) were quantified with the colocalization module of Volocity 5.2.1. in non-permeabilized cells at 4°C (white) and 37°C (grey), in comparison to permeabilized cells (black). Data in bar graphs are expressed as the mean+SE (*R* value) of triplicate sections from 10 donors. Asterisks (*p<0.01) indicate significant differences between the *R* values of non-permeabilized cells compared to permeabilized cells.

To investigate whether the tetraspanin-enriched VCCs in HCs can be accessed from the plasma membrane, we tested the ability of antibodies against CD9 and CD81 to stain the compartments before permeabilization. Staining was performed at 4°C to reduce membrane movement and phagocytosis, while labelling at 37°C mimics *in vivo* conditions and allows for active uptake. HIV-1_BaL_ infected HCs were immunolabelled at 4°C and 37°C prior to fixation. To control for antibody specificity, we repeated all immunofluorescence experiments with monoclonal anti-TfR, an unrelated protein that does not colocalize with Gag in HCs (Supplementary file 2). At 4°C, tetraspanins were limited to the plasma membrane of infected HCs, while Gag was readily detected in the VCCs (Figure 5a). In contrast, HIV-infected HCs first immunolabelled with antibodies against CD9 and CD81 at 37°C and then permeabilized displayed prominent labelling and colocalization with Gag within the VCCs (Figure 5b). We compared the degree of colocalization between the tetraspanins and Gag in HIV-1-infected HCs immunolabelled at 4°C and 37°C prior to fixation, using cells from individual donors (Figure 5c). Unlike what we have previously shown in MDMs [13], these results confirm that VCCs within primary HCs are accessible to antibodies in the external environment.

### HIV-1-neutralizing antibodies can access VCCs and limited HIV-1 replication in HCs in an FcγRI-dependent manner

**Figure 6:**
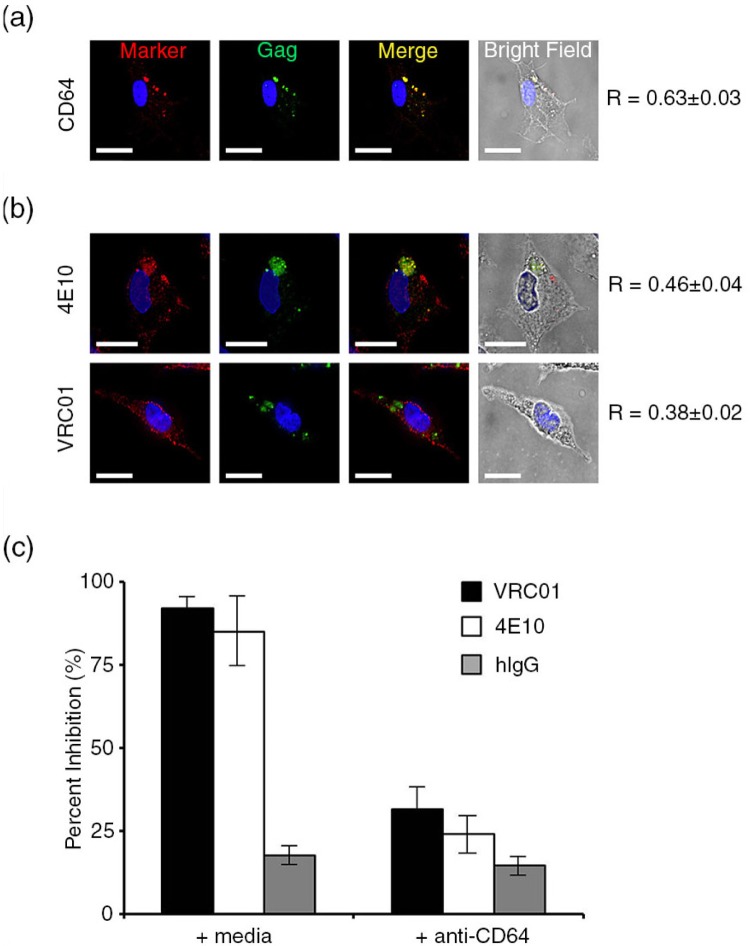
HIV-1-neutralizing antibodies access VCCs and inhibit HIV-1 replication in HCs in an FcγRI-dependent manner. (a) Human HCs were infected with HIV-1_BaL_ for three days. Infected HCs were immunolabelled at 37°C prior to fixation/permeabilization and labelled with primary antibodies against (a) FcγRI (red, anti-CD64), (b) 4E10 (red) and VRC01 (red). Labelled HCs were then permeabilized and immunolabelled for HIV-1 Gag (green). Scale bar=7 µm. Image acquisition was performed with an Applied Precision Deltavision deconvolution microscope. Sections shown represent a minimum of 30 cells for each condition from eight donors. Colocalization and partial colocalization *R* values (using Pearson's correlation) were quantified with the colocalization module of Volocity 5.2.1. (c) Neutralization activities of anti-HIV-1 neutralizing antibodies, VRC01 and 4E10, were determined in HCs at day 10 post-infection. Infected HCs were exposed to bNAbs alone. In competition experiments, HIV-1-infected HCs were also pre-incubated with the monoclonal antibody against FcγRI (anti-CD64). The percent of inhibition is defined as the reduction of p24 release in the supernatant of Ab-treated HIV-1-infected HCs compared with the control untreated HIV-1-infected HCs. Data in bar graphs are expressed as the mean+SE (% inhibition) of triplicate sections from eight donors. Asterisks (*p<0.01) indicate values that are significantly higher in VRC01- and 4E10-treated HCs compared to hIgG-treated controls. Symbols (*p<0.01) indicate significant differences due to the addition of anti-CD64.

It is unknown whether infected HCs are exposed to the maternal HIV-1 immune response *in utero*. To examine the role of bNAbs at the foeto-maternal interface, we administered anti-HIV-1 gp120 and gp41 antibodies (VRC01 and 4E10, respectively) at 37°C. HIV-1-infected HCs were also incubated with the monoclonal antibody against FcγRI (CD64), a receptor abundantly expressed on HC with great affinity for IgG [27,28]. HIV-1-infected HCs, first immunolabelled with antibodies against FcγRI (CD64) at 37°C and then permeabilized, displayed very specific FcγRI labelling within the VCCs (HIV-1 Gag colocalization *R* value of 0.63±0.03) (Figure 6a). In addition, 4E10 showed a strong pattern of colocalization with Gag (*R* value of 0.46±0.04), while VRC01 colocalization with Gag was present but less precise (*R* value of 0.38±0.02) (Figure 6b). However, the degree of colocalization was substantial for both bNAbs, compared with the anti-TfR controls (HIV-1 Gag colocalization *R* value of 0.2±0.03) (Supplementary file 2). To test the neutralizing activity of VRC01 and 4E10 in infected HCs, cells were productively infected overnight. Virus was removed, and cells were subsequently treated with the HIV-1-specific bNAbs. On day 4, the antibodies were removed, and viral replication was monitored and analyzed on day 10. Exposure to VRC01 and 4E10 following viral uptake strongly inhibited HIV-1_BaL_ replication in HCs (Figure 6c). These *in vitro* studies demonstrate that internal accumulations of HIV-1 by HCs may be accessible and permissive to the inhibiting activities of HIV-1-specific bNAbs.

To determine the role of FcγRI in antibody uptake, we performed antibody competition experiments with purified monoclonal FcγRI antibodies. HIV-1-infected HCs were incubated with FcγRI antibody prior to the addition of bNAbs (Figure 6c). Control virus was incubated with media or non-HIV-1-specific human IgG to control for Ab specificity. The inhibitory activity of the bNAbs was reduced significantly after blockage of FcγRI, which demonstrates that FcγRI may be largely involved in NAb entry.

## Discussion

VCCs have been identified in HIV-1-infected MDMs and harbour large numbers of mature virus particles [29,30]. It is not clear whether VCCs are susceptible to the effects of bNAbs. In the current study, we characterize a compartment where HIV-1 assembles and accumulates within placental macrophages. Our group and others have reported that HCs can be productively infected and exhibit reduced ability to replicate HIV-1 *in vitro*, in comparison to primary macrophages [6,31,32]. However, the intracellular sites of assembly and accumulation have never been examined but are important to characterize given the pivotal role of the placenta in offsetting (mother-to-child transmission) MTCT of HIV-1.

HCs were first described in the 1980s [19,21] and are found in stroma adjacent to the trophoblast and foetal capillaries [20,33]. They have been postulated to serve as a line of host defence, stromal maturation and villous development [19,33,34]. Morphological assessment of HCs at day 0 prior to HIV-1 infection revealed a predominance of rounded cells with CD9 in distinct intracellular compartments similar to those found in uninfected MDMs [12]. However, following culture for two days, more elongated (spindle-like) cells evolved. In contrast, MDMs demonstrate a rounded appearance post-infection and maintain intracellular VCCs for long periods of time. Despite morphological differences, HCs are classified as macrophages [20].

Tetraspanins are often recruited to sites of HIV-1 budding in virus-producing cells [35], and studies have shown that HIV-1 favours compartments enriched with tetraspanins [36]. Similar to HIV-1-infected MDMs, viral assembly sites within HCs are specifically marked by the tetraspanins CD9 and CD81, along with less precise staining by CD63 and the lysosomal markers, LAMP-1 and LT-Red. CD9 and CD81 were highly concentrated along with Gag. CD63 and LAMP-1 were also associated with compartmentalized HIV-1; however, the majority of cellular CD63 and LAMP-1 were found in an extensive network of vesicles throughout the cytoplasm that were CD9/CD81/Gag-negative. Our data show that HC VCCs are not specifically endosomes, but rather viral accumulation sites marked by the tetraspanins CD81 and CD9. In addition, we observed HIV-1 accumulation in acidic compartments stained with LT-Red. The endocytic pathway is characterized by its progressive acidification, which promotes activation of degradation enzymes [37]. HIV-1 is a fragile virus sensitive to low pH and proteases; therefore in infected MDMs, HIV-1 has evolved strategies to inhibit acidification of these endosomal compartments [10]. However, our results suggest that upon uptake in HCs, HIV-1 may be sequestered in acidic compartments, which could represent a dead-end for HIV-1 infection at the foeto-maternal interface. By characterizing the molecules involved in the assembly of HIV-1 in HCs, novel targets may be identified for potential therapeutic intervention.

Macrophages represent viral reservoirs in individuals with chronic HIV-1 infection and accumulate virus within an intracellular compartment where they remain infectious for long periods of time [14]. Recent observations describe tubular structures connecting the VCCs of HIV-1-infected MDMs to the cell surface with insufficient diameter for virion release [38]. Small channels linking the VCC in macrophages to the plasma membrane were first identified using a membrane-impermeant dye ruthenium red [29]. In addition, our recent findings indicate that the majority of VCCs in human MDMs are effectively closed compartments, inaccessible to antibodies, the cell surface label cationized ferritin, or low-molecular-weight molecules [13]. In this study, we quantified the accessibility of the intracellular VCC in primary HCs to entry of low-molecular-weight dextrans. With dextrans, we were able to show strong colocalization with Gag in intracellular compartments in unfixed cells at 4°C and 37°C. These results indicate that the majority of VCCs in HCs are accessible to small molecules, which may occur passively without active uptake.

Inaccessible VCCs found in MDMs potentially support the role of macrophages as reservoirs, where sequestered virus in endosomal compartments are not exposed to NAbs or antiretroviral drugs [25]. Intriguingly, our study provides strong supporting evidence that VCCs within HCs are accessible to surface administered antibodies, which may occur *in vivo*. These results are based on the usage of antibodies targeting the compartment (CD9, CD81, and CD64 (FcγRI)) or NAbs against HIV-1 (VRC01 and 4E10). Antibody uptake was reduced at 4°C; however, antibody colocalization with Gag was very pronounced at 37°C, indicating active antibody capture. 4E10 and VRC01 have been widely studied for their broadly neutralizing activity in HIV-1-infected blood-derived cells [39,40]. In macaques, passive immunization with bNAb combinations, including 4E10, was shown to be a promising approach to prevent MTCT [41]. In the current study, our data show that HCs sequester HIV-1 and allow 4E10 and VRC01 to access the VCCs, thus limiting intracellular viral replication thereby potentially serving as a potent antiviral mechanism at the foeto-maternal interface *in vivo*.

Recently, an FcγR-dependent mechanism of HIV-1 inhibition was detected in antigen-presenting cells [42–44]. Here we report that in HCs, VRC01 and 4E10 are largely dependent on FcγRI (CD64). FcγRI has a high affinity for IgG and is largely expressed at the cell surface of HCs [27,28]. FcγRI and DC-SIGN have both been shown to efficiently capture NAbs and immune complexes in DCs [45]. In addition, a recent study detailing intrauterine CMV infection describes Fc-receptor-mediated transport of maternal antibodies and virus-Ab complexes across the trophoblast, with subsequent capture by HCs in the villous core [46]. These are internalized via receptor-mediated endocytosis and transported to an early endosomal compartment [45]. The inhibitory activity of 4E10 and VRC01 was reduced significantly after blockage with FcγRI. This data suggest a critical role for Fc-FcγRI interactions in antiviral functions of bNAbs, which may facilitate reducing HIV-1 replication and dissemination *in vivo*. Further studies are necessary to understand the clinical implications of bNAbs to inhibit HIV-1 replication at the foeto-maternal interface.

A potential limitation of the current study is the selection of placentae at the end pregnancy. While *in utero* transmission of HIV-1 is only 7%, evidence shows this transmission occurs late in gestation [47,48]. Although studies have identified viral infection in foetuses as early as eight weeks [49], the likelihood of *in utero* transmission during the first or second trimester is rare [50]. This is balanced by more recent reports which demonstrate that HCs are more abundant in early pregnancy compared to the third trimester [51]; therefore, consideration should be given to include early gestation HCs in future studies.

## Conclusions

We demonstrate that HCs sequester HIV-1 in unique tetraspanin-positive VCCs that are readily accessible to exogenously applied NAbs, including VRCO1 and 4E10. Collectively, these results provide evidence that placental HCs facilitate sequestration of HIV-1, and may serve as a site for intracellular neutralization or antiretroviral drug access to offset MTCT of HIV-1 *in vivo*.

## Supplementary Material

Placental Hofbauer cells assemble and sequester HIV-1 in tetraspanin-positive compartments that are accessible to broadly neutralizing antibodies

Placental Hofbauer cells assemble and sequester HIV-1 in tetraspanin-positive compartments that are accessible to broadly neutralizing antibodies
