# Supplementary figures and images for: Placental Hofbauer cells assemble and sequester HIV-1 in tetraspanin-positive compartments that are accessible to broadly neutralizing antibodies
Source: J Int AIDS Soc. 2015 Jan 22;18(1):19385. doi: 10.7448/IAS.18.1.19385 (PMC4308659; doi:10.7448/IAS.18.1.19385)

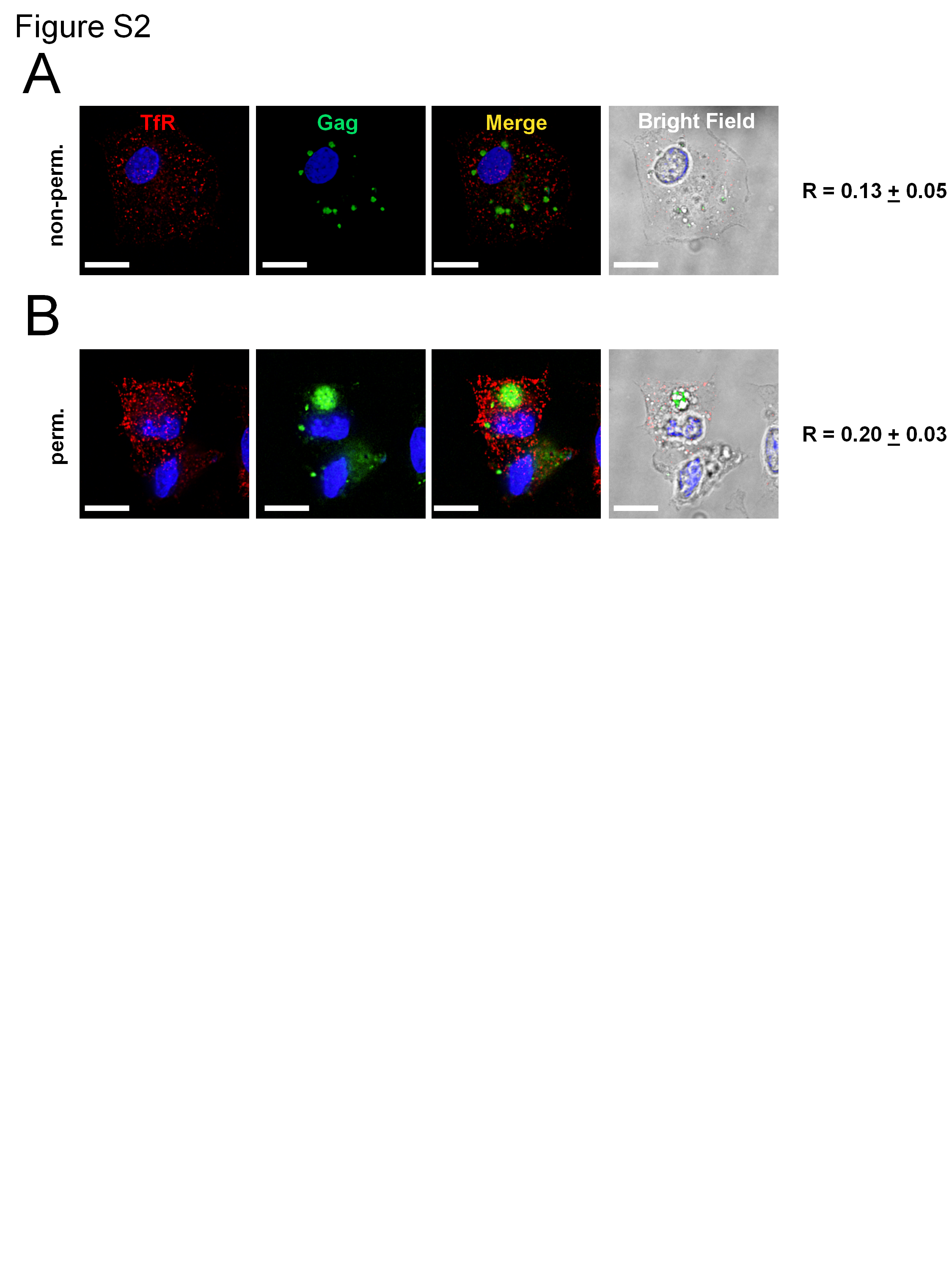

Supplement: Placental Hofbauer cells assemble and sequester HIV-1 in tetraspanin-positive compartments that are accessible to broadly neutralizing antibodies [file JIAS-18-19385-s001.tif]

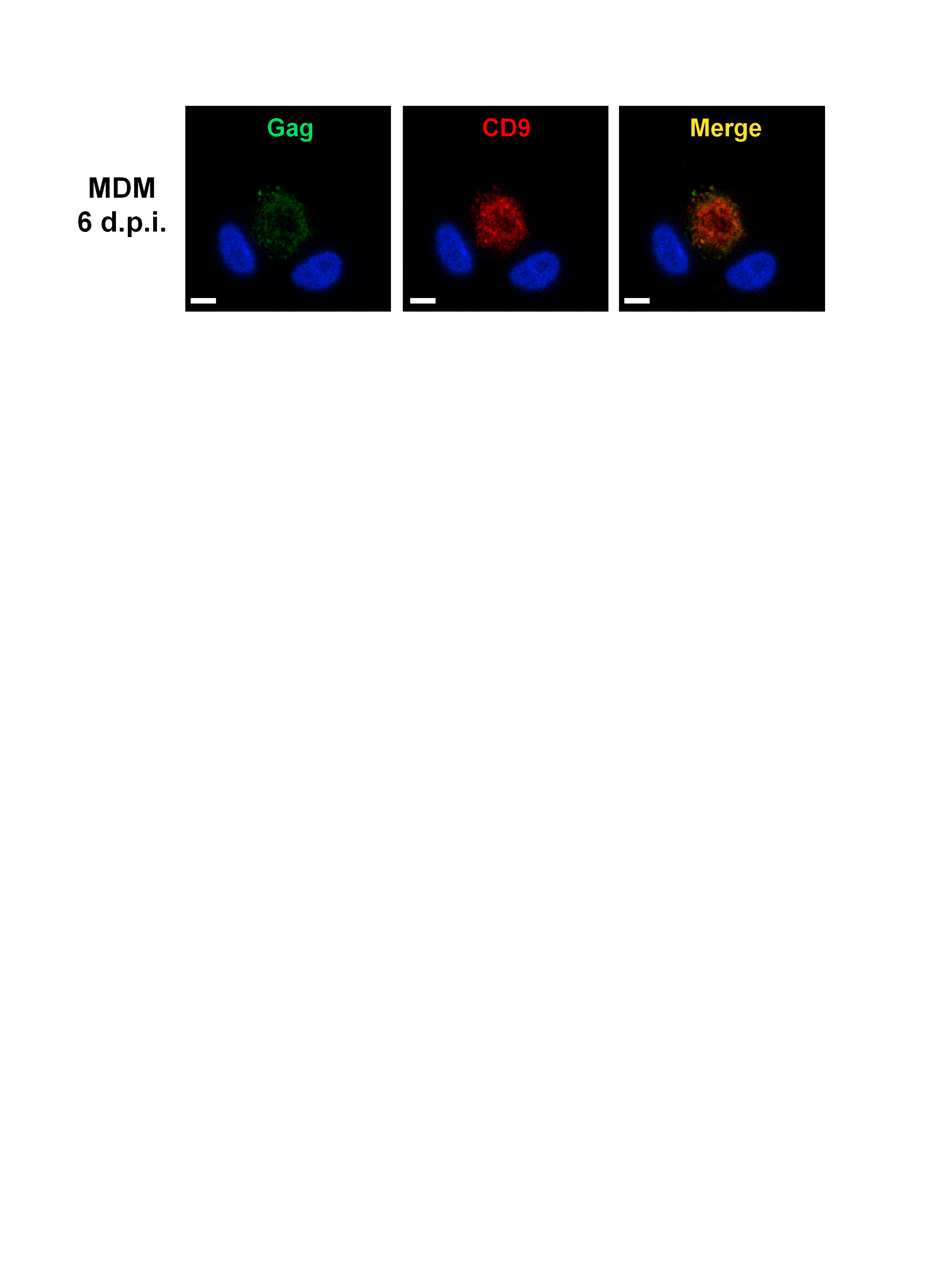

Supplement: Placental Hofbauer cells assemble and sequester HIV-1 in tetraspanin-positive compartments that are accessible to broadly neutralizing antibodies [file JIAS-18-19385-s002.tif]
